# Comparative Analysis of Methodological Aspects of the Study of Extracellular Vesicles and Extracellular Mitochondria: From Isolation to Internalization

**DOI:** 10.3390/cimb48020217

**Published:** 2026-02-16

**Authors:** Natalia Yunusova, Dmitry Svarovsky, Evgenya Kaigorodova, Alexey Dobrodeev, Virab Sisakian, Svetlana Tamkovich

**Affiliations:** 1 Cancer Research Institute of the Tomsk National Research Medical Center of the Russian Academy of Sciences, 5, Kooperativny Str., 634009 Tomsk, Russia; svarovsky.d.a@gmail.com (D.S.); zlobinae@mail.ru (E.K.); dobrodeev@oncology.tomsk.ru (A.D.); 2 Department of Biochemistry and Molecular Biology with the Course of Clinical Laboratory Diagnostics, Siberian State Medical University, 2 Moskovsky Tract, 634050 Tomsk, Russia; 3 Department of Obstetrics and Gynecology, Institute of Medicine and Medical Technologies, Novosibirsk State University, 2, Pirogov Str., 630090 Novosibirsk, Russia; v.sisakian@g.nsu.su; 4 Institute of Oncology and Neurosurgery of the National Medical Research Center Named ac. E.N. Meshalkin, 15 Rechkunovskaya Str., 630055 Novosibirsk, Russia; tamkovich_sn@mechalkin.ru

**Keywords:** extracellular mitochondria, mitochondria-rich extracellular vesicles, exosomes, mitochondrial transfer, mitochondrial proteins, mitochondrial DNAs and RNAs, internalization, platelet concentrate, stem cells

## Abstract

Mitochondrial transfer in mammals has been proven to occur both under physiological conditions and during pathological conditions. It has been shown that neighboring cells can exchange mitochondria via nanotunnel tubes. However, there is evidence that free mitochondria, as well as whole mitochondria and individual mitochondrial fragments, can be transported between cells within extracellular vesicles (EVs). This review discusses the methodological aspects of isolation and a minimal set of methods for characterizing mitochondria-rich EVs (mitoEVs), as well as methodological approaches for studying the nucleic acid, protein, and lipid composition. It has been shown that mitoEVs, as well as extracellular mitochondria, contain a characteristic set of nucleic acids of mitochondrial origin. First and foremost, the dominant fraction of mitochondrial nucleic acids is mitochondrial DNA (mtDNA), a circular double-stranded molecule approximately 16.6 thousand base pairs in length. The mechanisms involved in EV internalization include clathrin-dependent endocytosis, caveolin-dependent endocytosis, raft-mediated endocytosis, and macropinocytosis. Mitochondrial-enriched autologous and xenogeneic EVs are thought to be internalized by similar mechanisms. The review also presents the main sources (stem cells, platelet concentrate, peripheral blood mononuclear cells) for obtaining mitochondria-rich EVs for therapeutic purposes.

## 1. The Presence of EVs and Extracellular Mitochondria in Human Circulation

In this review, we tried to use the nomenclature of extracellular vesicles (EVs) recommended by the MISEV 2023 experts, which proposes to characterize vesicle types according to (a) physical characteristics such as size (small EVs <200 nm and medium/large vesicles from 200 to 2000 nm) or density; (b) biochemical composition (e.g., CD9+ EVs, GFAP+ EVs); (c) description of the conditions of EV production or their cellular origin (e.g., hypoxic EVs, aproptotic bodies) [1]. According to these recommendations, small EVs should not be called exosomes unless their endosomal origin is proven. Consensus regarding specific markers of EV subtypes, such as “exosomes” of endosomal origin and “ectosomes” (microparticles/microvesicles) derived from the plasma membrane, has not yet been reached. However, many researchers working in the field of EVs research continue to use the terms “exosomes,” “microvesicles,” and “microparticles”, so we tried to adapt the available literature to the MISEV 2023 expert recommendations [2]. Extracellular vesicles (EVs) of various sizes (from 40 to 1000 nm) and compositions are well identified in blood and other human biological fluids (saliva, urine, cerebrospinal and tear fluids, breast milk, ascites) as round membrane objects of the nanometer range containing extracellular DNA, mRNA fragments and non-coding RNA, as well as proteins and lipids. Culture fluids also contain significant amounts of classical EVs [3,4].

Mitochondria, as complex intracellular organelles with a diameter of 0.5–3 μm and surrounded by a double membrane, have been fairly well studied. The outer mitochondrial membrane (MOM) is adjacent to the cytoplasm and contains many proteins involved in the normal functioning of the organelle, its interactions with other structures, and intracellular transport. MOM has a porous structure due to the presence of porins, which allows ions and small uncharged molecules to penetrate into the intermembrane space [5]. The inner mitochondrial membrane (MIM) forms cristae and provides a surface for components of the electron transport chain. Molecules and proteins penetrate the MIM exclusively with the help of specific transport proteins. The mitochondrial matrix is place where ketogenesis, amino acid metabolism, steroid hormone synthesis, heme synthesis, and the tricarboxylic acid cycle occur. The intermembrane space is located between the outer and inner membranes. The structure of the MIM creates an electrochemical gradient by pumping protons from the matrix into the intermembrane space, which is used by ATP synthase during oxidative phosphorylation to generate ATP [6]. Mitochondria possess their own circular DNA (mtDNA) approximately 16,000 base pairs long, consisting of light and heavy chains. Most of the coding sequences are localized in the heavy chain. The mitochondrial genome encodes 37 transcripts: 13 subunits of the respiratory chain (ETC) proteins, 2 rRNAs, and 22 mRNAs. Almost all mitochondrial proteins are encoded by the nuclear genome, synthesized in the cytoplasm, and transported to the mitochondria by translocase proteins localized on both the outer and inner membranes. The human mitochondrial genome encodes only 13 essential protein subunits—representing a small fraction (1%) of the over 1100 total mitochondrial proteins—which are crucial for oxidative phosphorylation. These 13 mRNAs are translated by specialized mitochondrial ribosomes within the matrix [7].

For the first time in 2004, Rustom A.C. et al. described specific structures-tunnel nanotubes (TNTs), through which intercellular organelle transport occurred [8]. Since about 2012, intensive research has been conducted to study the intercellular transfer of mitochondria through TNT in various cell cultures, in vitro/in vivo models simulating immunoregulatory interactions, intercellular interactions in the musculoskeletal system, stroke, heart attack, various neurodegenerative diseases, stromal-tumor relationships, the formation of chemo- and radioresistance, resistance to targeted drugs [9,10,11,12,13,14]. TNTs are membrane protrusions in the form of tubes up to 1500 nanometers in diameter that form between the plasma membranes of adjacent cells. Thus, due to the large diameter of the tubes, many organelles, including mitochondria, are translocated from cell to cell via TNTs [15,16]. This mechanism of mitochondrial exchange between cells has been well confirmed in various cell cultures. However, it is believed that the main mechanisms leading to the appearance of cell-free mitochondria in circulation are their transport as part of EVs (mainly as part of large EVs), as well as the process of extrusion (pushing out) of mitochondria from the cell [17,18,19,20].

Literary data indicate the existence in circulation of both free mitochondria and functionally competent mitochondria as part of EV, as well as EV with individual mitochondrial components (mtDNA, mitochondrial proteins) [17,18,21,22]. However, the majority of circulating EVs are mitochondria-free vesicles. The proportion of mitochondria-enriched vesicles is significantly higher in EVs larger than 300 nm [19,20,21]. This is understandable, because it has been shown that intracellular mitochondria, although significantly variable in size, are structures larger than 500 nm. The biogenesis of medium/large EVs has not been fully studied, but it is known that, the formation of medium/large EVs in breast cancer cells involves the actin-myosin mechanism and the small GTPase ARF6, which recruits extracellular signal-regulated kinase (ERK) to the plasma membrane, followed by ERK-mediated phosphorylation and activation of myosin light chain kinase (MLCK). MLCK activation induces actomyosin contractions at the neck of these structures, which leads to budding and release of medium/large EVs from the plasma membrane into the extracellular space. Dynamic light scattering data indicate that the size of the released EVs ranged from 300 to 900 nm [23,24].

The process of mitochondrial sorting into microvesicles is currently poorly understood. Data obtained primarily from brain endothelial cells indicate a significant role in this process for a protein involved in mitochondrial fission, dynamin-related protein 1 (DRP1), as well as a protein involved in mitochondrial attachment to the cytoplasmic membrane, mitofusin-1. Under certain conditions (stress, serum-free culture media, initially high levels of metabolism and mitochondrial synthesis in cells, specific immune cell inducers), normal division of intracellular mitochondria in the region of membrane contact sites can result in the formation of small, fully functional mitochondria, which then attach to the plasma membrane (possibly fuse with it) and are subsequently secreted as large EVs. Most studies indicate that this process is associated with the suppression of mitophagy [20,25].

## 2. Methodological Aspects of the Study of EVs and Extracellular Mitochondria

### 2.1. Isolation of EVs and Extracellular Mitochondria and Their Minimal Characterization

For various purposes, EVs can be isolated from various sample types using several approaches, including ultracentrifugation, gradient centrifugation, ultracentrifugation combined with ultrafiltration, precipitation using polyethylene glycol and other polymers, immunoprecipitation, gel chromatography, and others. Most researchers consider the mitochondria-rich EVs (mitoEVs) fraction to be one subset of the total circulating EV pool, and therefore, similar approaches are generally used. Most studies on this topic indicate that exosomes and so-called small EVs do not contain intact mitochondria; therefore, to obtain mitochondria-enriched EV fractions, ultrafiltration of supernatants of biological and culture fluids through filters smaller than 200–220 nm is not recommended [19,21,25].

However, specific methods for isolating mitochondria-enriched EVs are limited because their detailed characteristics, such as size, surface markers, and density, remain unclear. Differential centrifugation or centrifugation combined with ultracentrifugation without the use of additional sedimentation agents is one of the most commonly used methods for isolating such vesicles. Typically, authors limit themselves to three rounds of centrifugation of blood plasma or other biological or cultural fluids. The first centrifugation is typically performed at 400–800 g for 5–10 min to remove cells. The supernatant is then centrifuged for 10–15 min at 1500–2500 g to remove cellular debris, and the resulting supernatant is then centrifuged at 16,000–16,500× *g* for 10–30 min to obtain the microvesicle fraction (all centrifugations should be performed at 4 °C). If mitochondrial fragment-rich small EVs are to be studied, ultracentrifugation of the samples at 100,000–118,000× *g* is necessary, with the duration of ultracentrifugation varying from 30 min to 3.5 h [3,4,18,19,21]. It is important to understand that the resulting fractions will be mixed, and only a portion of these EVs will contain either intact mitochondria or their fragments.

We believe that approaches based on specific immunoadsorption should probably be used to enrich the mitoEVs fraction. Several authors report the use of immunoprecipitation for the specific sorption of mitoEVs and freely circulating mitochondria. In this case, antibodies to MIM proteins of mitochondrial cytochrome c oxidase 2 subunit MT-CO2, to MAPL-mitochondrial anchored protein ligase, to inner membrane translocase TIM 23, to MOM translocases-TOM22 and TOM20 are most often used [18,21,26]. However, it was subsequently shown that both the total EV fraction and the immunoprecipitated subfractions, as well as the EV fraction obtained after depletion of large EVs (ultrafiltration through a 0.22 nm filter) contain both mitochondrial 16S rRNA and cytoplasmic 18S rRNA, mitochondrial DNA, as well as classical EV markers (CD9, CD81, annexin and Alix), which may indicate a crossover of mitochondrial and vesicle biogenesis [18,21,26].

D’Acunzo et al. developed a new approach involving ultrafiltration (0.22 μm) and iodixanol-based high-resolution density gradient centrifugation to isolate mitovesicles from brain tissue [27]. This approach will allow the detection of a fraction of very small EVs (mitovesicles) in the total fraction of EVs. Markers of microvesicles, exosomes, and endocytosis were not detected in this fraction. The mitovesicle fraction contained a high proportion of double- and triple-membrane electron-dense vesicle-like structures and was uniquely enriched in mitochondrial proteins of MOM, MIM, and mitochondrial matrix, as assessed by antibodies to the voltage-dependent anion channel (VDAC), cytochrome c oxidase subunit 4 (COX-IV), and pyruvate dehydrogenase E1 component alpha subunit (PDH-E1). However, this approach is currently only applied to brain tissue and melanoma metastasis tissue, and its applicability to other types of biological samples requires further study [21,27].

Methods for general characterization of EVs (morphology, size, and surface markers) were proposed in the Minimum Information for Studies on Extracellular Vesicles 2023 (MISEV 2023) guideline and include nanoparticle tracking analysis, dynamic light scattering, electron microscopy, and cryo-electron microscopy (cryo-EM) [1]. Evaluation of EV morphological characteristics using traditional electron microscopy based on chemical fixation and negative contrast is accompanied by the risk of distorting the native structure of vesicles. Cryo-EM avoids these limitations by visualizing EVs in a vitrified state, without dehydration and the use of contrast agents [28]. As a result, most EVs exhibit a spherical shape and a clearly defined membrane structure, in contrast to the artifactual “cup-shaped” morphology characteristic of standard EM. Cryo-EM confirms the preservation of the characteristic ultrastructure of mitochondria, including the double membrane and cristae, as well as their functional competence after transfer to recipient cells [29]. The use of cryo-EM additionally allows one to observe the interaction of mitochondria with vesicle membranes and reconstruct the stages of their secretion [30]. Cryo-EM of vesicles isolated during culture of visceral and subcutaneous adipose tissue from obese patients revealed not only single-membrane vesicles but also double-membrane vesicles, as well as multilamellar and granular vesicles. The authors also found that vesicles with internal membrane structures (multilamellar and granular vesicles) were characteristic of visceral adipose tissue, but not subcutaneous adipose tissue. Although the authors did not perform a proteomic analysis of such vesicles and did not stain them with dyes specific for mitochondrial membranes, visually such vesicles are similar to mitoEVs described in other studies [17,27,31]. Detection of surface tetraspanins by flow cytometry and immunoblotting is also a necessary step in characterizing the identified structures. In our opinion, a certain contribution to the evidence for the isolation of mitochondria-rich EVs is made by the assessment of the metabolic activity of such vesicles (the presence of ATP synthase activity, confirmation of the presence of respiration using inhibitors and activators [17,21].

Staining of isolated non-fixed vesicles or extracellular mitochondria with the cationic carbocyanine dyes Mito Tracker Red or Mito Tracker Green, which are able to penetrate the plasma membrane and selectively accumulate in active mitochondria depending on their membrane potential, allows detection of such structures by flow cytometry or immunofluorescence [18,32]. Active transport of positively charged protons across the MIM results in an internal negative charge—the mitochondrial transmembrane potential. The proton gradient is then used by ATP synthase to synthesize ATP. The net negative charge in healthy mitochondria is maintained at around −180 mV, which can be determined by staining cells with positively charged dyes such as tetramethylrhodamine ethyl ester (TMRE). TMRE emits red fluorescence, which can be detected by flow cytometry or fluorescence microscopy, which is also used to detect mitoEVs or free mitochondria [33]. A summary of approaches to isolating EVs enriched in mitochondria or mitochondrial content is presented in Table 1.

### 2.2. Detailed Characterization of the Nucleic Acid Composition of Mitochondria-Enriched Extracellular Vesicles and Extracellular Mitochondria

MitoEVs, as well as extracellular mitochondria, contain a characteristic set of nucleic acids of mitochondrial origin. First and foremost, the dominant fraction of mitochondrial nucleic acids is mtDNA, a circular double-stranded molecule approximately 16.6 thousand base pairs in length [34]. Intact mitochondria can be detected in blood through identification of extracellular full-length mtDNA. An intact mitochondrial genome can be released from cells either as part of a whole mitochondrion or within EVs [17]. EVs become enriched in mtDNA under certain conditions; in particular, a significant amount of vesicular mtDNA has been detected in the serum of patients with colorectal cancer, with its concentration increasing in patients with more advanced stages of tumor progression [35]. Qualitative analysis of this mtDNA demonstrated its integrity: using long-range PCR amplification, the authors identified three overlapping regions corresponding to full-length vesicle-derived mtDNA. This indicates that EVs are capable of transferring entire mtDNA molecules rather than only fragmented sequences. Functionally, mtDNA within extracellular vesicles can act as an antigen, since its circular structure is evolutionarily similar to bacterial DNA, and its presence can induce inflammatory responses in recipient cells [36].

The amount of mtDNA in EVs changes under both physiological and pathological conditions. The content of vesicular mtDNA may decrease with aging: small EVs obtained from older donors contain less mitochondrial genetic material than EVs derived from young individuals [37]. Platelet activation also promotes active release of their own mitochondria into the extracellular space, both as intact organelles and as components of vesicles [38,39]. Platelets can therefore be considered one of the key sources of extracellular mitochondria and mtDNA: each platelet contains approximately four to six mitochondria involved in regulation of platelet function, and platelet activation leads to their release into the extracellular milieu [40].

It has also been shown that EVs may contain mitochondrial RNA (mtRNA), representing the pool of RNA transcripts derived from the mitochondrial genome. In extracellular vesicles isolated from the plasma of patients with Alzheimer’s disease, an increased relative level of mtRNA has been reported, mainly mRNAs encoding proteins of the mitochondrial electron transport chain (MT-ND1, MT-ATP6, MT-CO1, etc.), as well as mitochondrial ribosomal components (MT-RNR1 rRNA) [41]. It is likely that mitochondrial tRNAs may also be incorporated into EVs together with mRNAs, although their content is difficult to quantify. Nevertheless, recent studies confirm the presence of mitochondrial tRNAs in extracellular vesicles. In particular, a 2022 study by Austrian researchers demonstrated that EVs from urine and from renal proximal tubular epithelial cell cultures contain measurable amounts of non-coding mitochondrial RNA, including tRNAs, the levels of which changed upon in vitro modeling of chronic kidney disease through induction of inflammatory processes in these cells [42]. A separate category of interest comprises mitochondrial microRNAs (mitomiRNAs), which are translocated into mitochondria and are involved in regulation of mitochondrial genome expression. Although studies specifically identifying mitochondrial microRNAs in mitoEVs are scarce, it has been shown that the microRNA profile of EVs often reflects changes in mitochondrial function. For example, under mitochondrial stress in liver cells, accumulation of specific microRNAs (such as miR-146a) was observed both within mitochondria and in EVs secreted by these cells [43].

Differences in the nucleic acid composition of extracellular mitochondria and mitoEVs vesicles are determined by several key features. Extracellular mitochondria generally contain multiple copies of mtDNA and a complete set of mitochondrial RNAs, whereas vesicle-associated mitochondria can exist in two forms: mitochondria-enriched EVs (often referred to as mitoEVs), and smaller microvesicles (30–150 nm) that are unable to accommodate an entire mitochondrion. Such microvesicles may contain mitochondrial fragments, including portions of the matrix, membranes, and even mitDNA complexes, as described in the aforementioned study by D’Acunzo et al. [27].

Analysis of mtDNA and mtRNA in EVs requires modification of standard nucleic acid isolation and identification methods in order to increase sensitivity. This is due to the low abundance of mitochondrial nucleic acids and the need to distinguish them from nuclear counterparts. In many cases, standard real-time PCR is employed, often determining the ratio of mtDNA to nuclear DNA. This approach is also applied for identification of mitRNA: after isolation of total RNA from vesicles, reverse transcription is performed followed by quantification of mitochondrial gene transcripts. Digital PCR is successfully used to detect ultra-low concentrations of mtDNA in EVs, with sensitivity reaching detection of approximately 1000 mtDNA copies per microliter of sample [44]. Sequencing of vesicle-derived mtDNA, as performed in the aforementioned study by Guan B. et al. (2024), was also one of the key methodological approaches used to characterize the nucleic acid composition of EVs from patients with colorectal cancer [35].

### 2.3. Detailed Characterization of the Protein and Lipid Composition of Mitochondria-Enriched EVs and Extracellular Mitochondria

Various methods, such as proteomics and lipidomics, were used to globally determine the mitochondrial content of mitoEVs [21,27]. For example, a study of the proteome of EVs spontaneously secreted by neural stem cells derived from the subventricular zone of mouse brain revealed that these vesicles are enriched in mitochondrial proteins of the outer membrane, matrix, inner membrane, and ETC. Subsequent studies confirmed the presence of intact mitochondria, both as free organelles and encapsulated in EVs. The authors demonstrated that mitochondria released by neural stem cells have intact ETC complexes, preserve mitochondrial membrane potential and respiration [29].

In the work of O.R. Stephens et al. (2020), circulating mitoEVs from human plasma were sorted based on TMRE positivity for proteomic analysis. A total of 40 proteins were identified, among which were nine mitochondria-specific proteins and nine proteins putatively present in mitochondria, demonstrating the role of EVs in mitochondrial transport between cells. The majority of these vesicles were of endothelial origin, which is a distinctive feature of such vesicles, since the majority of vesicles from the total pool of human plasma vesicles have traditionally been of platelet origin [33,45]. However, such mitochondrial transfer was demonstrated only under conditions of cell-cell contact in cell cultures in the work of Stephens O.R. The authors believe that circulating mitochondria are capable of returning to cells, but there is some doubt about how long mitochondria maintain their function outside the cell, since most of the proteins required for mitochondrial function are encoded by the nuclear genome [33].

Our proteomic profiling of tumor-specific small EVs cargo revealed a significant enrichment of mitochondrial proteins in EVs obtained exclusively from tumor cells (nine cell lines), while such cargo was absent from non-tumorigenic breast epithelial cell lines (two lines). The identified proteins were predominantly associated with the oxidative phosphorylation system, with complex I subunits most frequently detected, while complex II components were completely absent. These results indicate a cancer-specific mechanism of mitochondrial protein incorporation into small EVs potentially reflecting altered energy metabolism and stress responses in malignant cells [22].

Detailed studies of the outer membrane proteins of such vesicles provide insight into their possible biogenesis and other valuable information, but they are relatively rare. To compare the expression of surface proteins isolated from EVs of five metastatic human melanoma tissues, three non-tumorigenic cell lines (HEK293T, TF1, and HMC1), and an MMSCs cell line, the cells were treated with sodium carbonate solution at pH 12, followed by membrane isolation. After treatment with a high-salt solution (1 M KCl) to remove proteins associated with the membrane via ionic bonds, the proteins in these samples were analyzed by mass spectrometry. The study revealed a high content of endoplasmic reticulum proteins and mitochondrial membrane proteins in vesicles from melanoma metastases compared to vesicles from non-tumorigenic cell lines. The bulk of the proteome of the membrane fraction of EVs from all cell lines was represented by classical vesicle markers (CD9, CD81, CD63, and flotillin), as well as plasma membrane proteins [21].

## 3. Internalization of EVs and Extracellular Mitochondria by Cells

The MISEV-2023 standard introduced a subchapter dedicated to the interaction of EVs with cells. It was shown that EVs can interact with target cells at different levels: plasma membrane binding or internalization. EVs contact the cell surface, which can be termed “EV binding.” In contrast, “EV uptake” encompasses several outcomes. This can mean EV fusion with the cell membrane and release of its contents into the cytoplasm. It can also mean internalization into endocytic and/or other intracellular compartments. The terms “EV internalization” and “EV uptake” are often interpreted as identical events [1]. Fluorescence microscopy and confocal microscopy are commonly used to visualize these events, allowing the identification of subcellular fluorescent events associated with cells, while flow cytometry primarily detects the “capture” of EVs without distinguishing between binding and uptake [46,47,48,49]. It should be noted that a rather low rate of EV internalization has been demonstrated for many effector cells, which dictates the need to carefully select the ratio vesicles/cells [46,49,50].

The endocytic mechanisms involved in EV internalization include clathrin-dependent endocytosis, caveolin-dependent endocytosis, and raft-mediated endocytosis. Macropinocytosis involves the uptake of EVs through the formation of large invaginations in the recipient cell membrane, from which vesicles subsequently bud off into the cytoplasm. Macropinosomes are large, necessarily fluid-filled, and may contain EVs trapped within this folded structure or free mitochondria. Macropinosomes do not carry specific molecules on their surface, unlike caveolae or clathrin-dependent endosomes. Formation of macropinosomes occurs with the participation of cytoskeletal contractions and is therefore subject to regulation by Rac GTPases and the PAK kinase family, requiring cholesterol and the activity of acidic lysosomal lipase. To study the detailed mechanisms of internalization, inhibitors of clathrin-dependent endocytosis (chlorpromazine), caveolin-dependent endocytosis (gynestein, nystatin, filipin, etc.), inhibitors of raft-dependent endocytosis, which destroy lipid rafts in various ways (for example, fumosin B1, methyl-beta-cyclodextrin, and simvastatin), inhibitors of macropinocytosis (EIPA, LY294002), mixed inhibitors (for example, dinasore), inhibitors of cholesterol absorption (for example, ezetimibe), inhibitors of lysosomal lipase (for example, lalistat 2) are most often used in cell cultures [51]. Mitochondrial-enriched autologous and xenogeneic EVs are thought to be internalized by similar mechanisms. However, it has been shown that intracellular mitochondria isolated using traditional methods are internalized by recipient cells primarily via macropinocytosis. Fluorescence microscopy and flow cytometry revealed suppression of mitochondrial transfer in the group with the macropinocytosis inhibitor EIPA compared to the group without EIPA. The same study also demonstrated that mitochondrial transfer was reduced by cytochalasin D (an actin polymerization inhibitor) and nocodazole (a microtubule assembly inhibitor), but not by chlorpromazine (an inhibitor of clathrin-mediated endocytosis) [52,53].

Traditional approaches to studying EV internalization are presented in many studies [19,46,53,54]. There are differences in vesicle internalization by blood leukocytes due to the age of the vesicle donor [54]. It has been previously shown that EV internalization is quite complex and is determined not only by the composition of EVs (the presence of tetraspanins, integrins, proteoglycans, lectins), but also by the properties of recipient cells (lipid composition of the cell membrane, representation of GPI-anchored proteins in the membrane, the presence of fibronectin, fibrinogen, laminins, ICAM-1, ICAM-2 on the cell surface, the state of the cytoskeleton proteins, the activity of other cellular processes). For example, it has been shown that macropinocytosis competes with other cellular pathways, such as cell migration, shape maintenance, phagocytosis, and pseudopodia formation for cytoskeletal resources and other cellular components, which also affects the efficiency of macropinocytosis [53,55,56]. The methodological aspects of studying the functionality of endogenous or exogenous mitoEVs, cell-free mitochondria isolated from blood plasma, or isolated intracellular mitochondria are beyond the scope of this article.

## 4. Methodological Aspects of Obtaining EVs Enriched with Mitochondrial Components for Therapeutic Purposes

Methodological issues related to obtaining EVs enriched with mitochondria and mitochondrial components remain poorly understood. These methodologies are currently being implemented at the level of studies on cell cultures or experimental animal models. The main sources of the total EV fraction and EVs enriched with mitochondrial components are multipotent mesenchymal stromal cells (MMSCs) of various origins (from bone marrow, adipose tissue, umbilical cord tissue, and umbilical cord blood), as well as unipotent tissue stem cells (e.g., neural stem cells) [10,29,57,58]. Induced pluripotent stem cells (iPSCs), which are usually obtained by transfection from peripheral blood mononuclear cells (PBMCs) using viral vectors, have some potential in this regard [59]. In particular, mitoEVs from induced cardiomyocytes were obtained in this way, which effectively restored the energetics of ischemic myocardium in a mouse model of myocardial infarction [19].

Another important source of mitochondrial-enriched EVs are available blood components, namely activated monocytes or PBMCs and platelet concentrate. It should be noted that these blood fractions are collected at blood transfusion stations and stored for a long time in blood biobanks, and the methods of their collection and storage are relatively standardized [18,39,60,61]. Red blood cell suspension does not appear to be a significant source of mitoEVs. EVs of red blood cell origin do not contain mitochondria, as red blood cells do not, although the concentration of isolated EVs is quite high when standardly prepared red blood cell suspensions are stored at 4 °C [62]. Modern blood service principles require the highest possible standardization of blood components to improve the quality of transfusion therapy and reduce the incidence of immune and infectious transfusion complications. Technical regulations for the production of blood components typically include: phenotyping of alloerythrocytes for erythrocyte antigens A, B, D, C, c, E, e, K, k, Cʷ; leukoreduction of erythrocyte-containing blood components and platelet concentrates; automated production of fresh frozen plasma and platelet concentrates; adherence to optimal screening times for markers of blood borne infections and quarantine of donor plasma. Thus, modern blood component production guarantees the safety of their use.

Summary data on sources of mitochondrial-enriched EVs for therapeutic purposes and approaches to their production are presented in Table 2.

**Figure 1:**
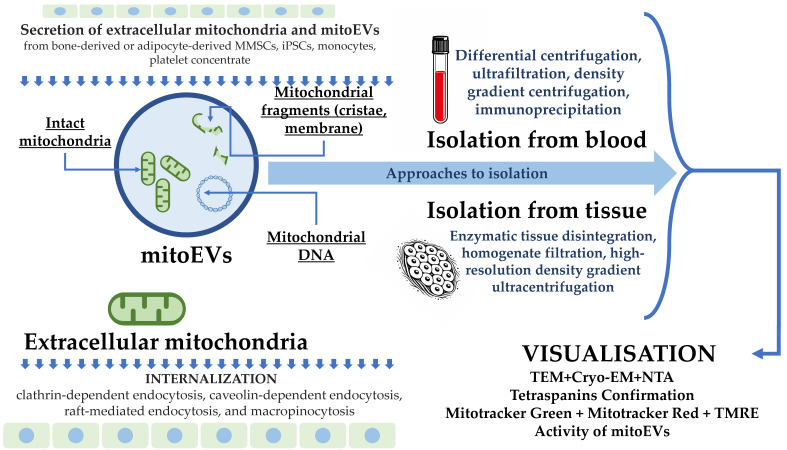
Isolation and characterization strategies for extracellular mitochondria and mitochondria-associated extracellular vesicles. Note: mitoEVs, mitochondria-associated extracellular vesicles; TEM, transmission electron microscopy; Cryo-EM, cryo-electron microscopy; NTA, nanoparticle tracking analysis; TMRE, tetramethylrhodamine ethyl ester; ETC, electron transport chain; MMSCs, multipotent mesenchymal stromal cells; iPSCs, induced pluripotent stem cells.

Isolation and characterization strategies for extracellular mitochondria and mitochondria-associated EVs are shown at Figure 1.

**Resume.** Mitochondrial transfer in mammals has been proven to occur both under physiological conditions and during pathological conditions. It has been shown that neighboring cells can exchange mitochondria via nanotunnel tubes. However, there is evidence that free mitochondria, as well as whole mitochondria and individual mitochondrial fragments, can be transported between cells within extracellular vesicles. The methodological aspects of isolation and a set of methods for characterizing mitoBB and extracellular mitochondria, as well as methodological approaches to studying the composition of nucleic acids, proteins and lipids are largely similar, but there are also certain differences. It has been shown that mitoEVs, as well as extracellular mitochondria, contain a characteristic set of nucleic acids of mitochondrial origin. First and foremost, the dominant fraction of mitochondrial nucleic acids is mitochondrial DNA (mtDNA), a circular double-stranded molecule approximately 16.6 thousand base pairs in length. The mechanisms involved in EV internalization include clathrin-dependent endocytosis, caveolin-dependent endocytosis, raft-mediated endocytosis, and macropinocytosis. Methodological approaches for studying detailed aspects of the internalization of mitoBB, autologous and xenogeneic mitochondria are continuously being improved. The review also presents the main sources (stem cells, platelet concentrate, peripheral blood mononuclear cells) for obtaining mitochondria-rich EVs for therapeutic purposes.

## Figures and Tables

**Table 1:** Summary of approaches to isolation mitochondria-enriched or mitochondrial-content-rich EVs.

Types of Samples	Approaches to Isolation of MitoEVs	Minimal Characteristics of MitoEVs	References
Conditioned medium	Differential centrifugation with/without ultrafiltration, sucrose density gradient centrifugation, ultracentrifugation with/without ultrafiltration, immunoprecipitation	TEM, Cryo-EM, NTA, confirmation of tetraspanins and Alix in the mitoEVs (flow cytometry or Western blotting); mitoEVs staining with specific fluorescent dyes Mitotracker Green, Mitotracker Red or TMRE; assessment of the metabolic activity of mitoEVs (ATP synthase activity, assessment of respiration by the rate of oxygen consumption using ETC complex inhibitors)	[3,4,17,18,19,20,22,25,26,27,29,31]
Blood and its components			
Samples of tumor metastases or tissue from experimental animals	Enzymatic tissue disintegration, homogenate filtration (50–70 μm filter size), homogenate ultrafiltration (220 nm filter size), high-resolution density gradient ultracentrifugation (on an iodaxinol gradient)		[21,27]

Note: TEM—transmission electron microscopy, cryo-EM—cryo-electron microscopy, NTA—nanoparticle tracking analysis, TMRE—tetramethylrhodamine ethyl ester, and ETC—electron transport chain.

**Table 2:** Summary data on sources and methods for obtaining mitochondria-enriched or mitochondrial-content-rich EVs for therapeutic use.

mitoEVs Sources	Manipulation of Cells to Produce mitoEVs	Advantages and Disadvantages of the Methods	Ref.
Bone marrow derived MMSCs	Approaches to obtaining MMSCs include enzymatic tissue disaggregation, filtration, centrifugation in Hanks’ saline solution, and resuspension in DMEM/F12 growth medium with fetal bovine serum. Seeding is performed on plastic Petri dishes, with non-adherent cells removed after 24 h. MMSC characterization includes typing for CD105, CD90, and CD73 with negative staining for CD45 and the ability to undergo osteogenic, adipogenic, and chondrogenic differentiation. MitoEVs is obtained from culture fluid during MMSC cultivation (see Section 2.1.).	Relatively accessible sources of obtaining MMSCs; there are unified protocols for obtaining and culturing MMSCs	[29,57,58]
Adipocyte -derived MMSCs			
MMSC of umbilical cord blood			
iPSCs	Promising non-viral methods for inducing pluripotency in human somatic cells include the piggyBac transposon carrying the transcription factors Oct4, Sox2, c-Myc, and Klf4, peptides fused via 2A, plasmid DNA, and recombinant transcription factors. A well-proven method for inducing pluripotency by transfecting somatic cells with mRNA for reprogramming factors (Oct4, Sox2, c-Myc, and Klf4) has been established. This method is safe, as the mRNA is completely degraded within the cell, and its in vitro synthesis does not involve the use of animal-derived materials. Molecular characterization of iPSCs is mandatory. MitoEVs is obtained from culture fluid using (see Section 2.1.).	It is possible to obtain patient-specific iPSCs. The advantages of mitoEVs when culturing iPSCs or specialized cells derived from iPSCs through directed differentiation over mitoEVs from MMSCs are unclear for therapeutic use. The complexity of iPSC production, the urgent need for maximum standardization of iPSC culture conditions, and safety concerns regarding the use of mitoEVs from iPSCs are unclear	[19,59,63]
PBMCs and activated monocytes	Simple, standardized, and inexpensive approaches to isolating and typing PBMCs (isolation on a Ficoll-Verografin gradient, magnetic immunoadsorption, and flow cytometry for typing). The need for standardized monocyte activation protocols for obtaining mitoEVs.	Available sources for obtaining mitoEVs. MitoEVs from blood components will carry class I and II histocompatibility antigens on their surface, therefore either careful selection of immunocompatible donors or the use of autologous blood components is required	[14]
Platelet concentrate	To induce the release of EVs, the platelet concentrate is subjected to several freeze-thaw cycles.	A variety of platelet concentrates, a wide range of laboratory equipment and consumables for platelet concentrate preparation, and the possibility of obtaining autologous platelet concentrates	[60,61]

Note: MMSCs—multipotent mesenchymal stem cells, iPSCs—induced pluripotent stem cells, and PBMCs—peripheral blood mononuclear cells.

## Data Availability

The data presented in this study are available request from the corresponding author.
